# Decolonisation of MRSA, *S. aureus* and *E. coli* by Cold-Atmospheric Plasma Using a Porcine Skin Model *In Vitro*

**DOI:** 10.1371/journal.pone.0034610

**Published:** 2012-04-27

**Authors:** Tim Maisch, Tetsuji Shimizu, Yang-Fang Li, Julia Heinlin, Sigrid Karrer, Gregor Morfill, Julia L. Zimmermann

**Affiliations:** 1 Department of Dermatology, Regensburg University Hospital, Regensburg, Bavaria, Germany; 2 Max Planck Institute for Extraterrestrial Physics, Garching, Bavaria, Germany; National Institutes of Health, United States of America

## Abstract

In the last twenty years new antibacterial agents approved by the U.S. FDA decreased whereas in parallel the resistance situation of multi-resistant bacteria increased. Thus, community and nosocomial acquired infections of resistant bacteria led to a decrease in the efficacy of standard therapy, prolonging treatment time and increasing healthcare costs. Therefore, the aim of this work was to demonstrate the applicability of cold atmospheric plasma for decolonisation of Gram-positive (Methicillin-resistant *Staphylococcus aureus* (MRSA), Methicillin-sensitive *Staphylococcus aureus*) and Gram-negative bacteria (*E. coli*) using an *ex vivo* pig skin model. Freshly excised skin samples were taken from six month old female pigs (breed: Pietrain). After application of pure bacteria on the surface of the explants these were treated with cold atmospheric plasma for up to 15 min. Two different plasma devices were evaluated. A decolonisation efficacy of 3 log_10_ steps was achieved already after 6 min of plasma treatment. Longer plasma treatment times achieved a killing rate of 5 log_10_ steps independently from the applied bacteria strains. Histological evaluations of untreated and treated skin areas upon cold atmospheric plasma treatment within 24 h showed no morphological changes as well as no significant degree of necrosis or apoptosis determined by the TUNEL-assay indicating that the porcine skin is still vital. This study demonstrates for the first time that cold atmospheric plasma is able to very efficiently kill bacteria applied to an intact skin surface using an *ex vivo* porcine skin model. The results emphasize the potential of cold atmospheric plasma as a new possible treatment option for decolonisation of human skin from bacteria in patients in the future without harming the surrounding tissue.

## Introduction

Eradication of multiresistant superbugs is one of the clinical challenges of the 21st century [1]. In addition the situation will further exacerbate, because the pipeline of new antibiotics is not fully straight. The Infectious Diseases Society of America (IDSA) highlights that, over the past several years, the number of new antibacterial drugs approved continues to decrease [2], [3]. Only three of the eight antibiotics approved within the last decade act towards a new mechanism of action, whereas all the others are only modifications of already known antibiotics. In parallel, the resistance of *S. aureus* increased substantially: Nowadays a total of ∼21% of *Staphylococcus aureus* in Germany are resistant to methicillin/oxacillin [4]. Klevens reported that the overall increase of methicillin-resistant *S. aureus* (MRSA) resistance was 3.1% per year in USA, based on a linear model from 1992–2003 [5]. Thus, there is great interest in new antimicrobial strategies to overcome the lack of new anti-invectives. Recently cold atmospheric plasma has demonstrated both bactericidal, virucidal and fungicidal properties due to the generation of reactive species and charged particles [6], [7], [8]. Numerous studies demonstrated the effectiveness of gas-discharge or cold atmospheric plasmas for killing especially planktonic microorganisms [7], [9], [10], [11]. Furthermore a successful disinfection of methicillin-resistant *Staphylococcus aureus* and *Staphylococcus epidermidis* biofilms was demonstrated using a remote non-thermal gas plasma [12].

In the context of decreasing numbers of new antibacterial drugs and increasing resistance of bacteria the effective disinfection of hospital surfaces is of major interest as an important factor in preventing hospital-acquired infections. First attempts were shown by Burts et al. who was able to demonstrate that disinfection of hospital pagers experimentally coated with MRSA could be achieved within 30 seconds of plasma treatment [13]. In general cold atmospheric plasma, which means less than 40°C at the point of application, can be produced in air in order to produce reactive molecules like atoms, ions and radicals. This chemical “cocktail” of reactive species can be used for different biomedical applications. The killing efficacies of plasma were in the same range as standard antimicrobial agents, up to 3–5 log_10_ steps [13]. However, to our knowledge, there are only few reports about the use of cold atmospheric plasma for decolonization of bacteria applied to vital skin surfaces [14], [15]. Lademann et al. for example used a plasma tissue tolerable plasma jet and achieved a reduction of the bacteria load of 94% using an *ex vivo* ear pig skin model [15]. However this plasma jet is able to disinfect only small skin areas (inhibition zone 2 mm) and was moved with an average velocity of 10 mm/sec on the skin surface.

For demonstrating the killing efficacies of new disinfecting agents an *ex vivo* porcine skin model was developed in our lab [16]. This model was used, as it serves as an alternative skin model for human skin based on many similarities regarding behaviour, composition, histological, physiological and permeability properties [17], [18]. The challenge of the antimicrobial plasma treatment is to find appropriate parameters which inactivate bacteria without harming the surrounding tissue. Therefore the present study was performed to evaluate the efficacy of two different cold-atmospheric plasma devices for decolonisation of ≥3 log_10_ steps (≥99.9%) of *S. aureus*, MRSA and *E. coli* on large skin areas when these bacteria were applied to an *ex vivo* porcine skin model.

## Materials and Methods

### Bacterial strains

The bacterial strains *Escherichia coli* (atcc 25922), methicillin-sensitive *Staphylococcus aureus* (atcc 25923) and two methicillin-resistant *Staphylococcus aureus* (MRSA: atcc BAA-44; attcc 43300) were grown aerobically at 37°C in Mueller-Hinton broth (Carl Roth GmbH+Co. KG, Karlsruhe, Germany). A 500 µl portion of an overnight bacteria culture (3 ml) was transferred to 50 ml of fresh BHI media and grown at 37°C on an orbital shaker. When the cultures reached the stationary phase of growth, the bacteria were harvested by centrifugation (200 *g*, 15 min), washed with 10 mM phosphate-buffered saline (PBS; Biochrom, Berlin, Germany) at pH 7.4 containing 2.7 mM KCl and 0.14 M NaCl and suspended in PBS at an optical density of 0.6 at 600 nm corresponding to 10^6–7^ bacteria ml^−1^.

### Preparation of pig skin

Fresh skin from six month old female pigs (breed: Pietrain) was obtained from a local slaughterhouse (Metzgerei Stierstorfer, Wenzenbach, Germany). The preparation was done as already published [16]. Briefly, the excised skin was washed with water, epilated with a dry razor and the adipose tissue beneath the dermis was removed with a scalpel. Then the skin was cut under sterile conditions into 2×2 cm^2^ pieces, placed in a Petri dish and embedded with Hepes Agar. The surface of the embedded skin was then incubated with 70% ethanol for 5 min to reduce the number of resident bacteria. After three washing steps with sterile PBS, a known number of bacteria (10^6–7^ ml^−1^ CFU) were applied to the skin and the treatment with cold atmospheric plasma was performed.

### Plasma devices

Two different cold atmospheric plasma devices were evaluated.

#### FlatPlaSter

**Figure 1:**
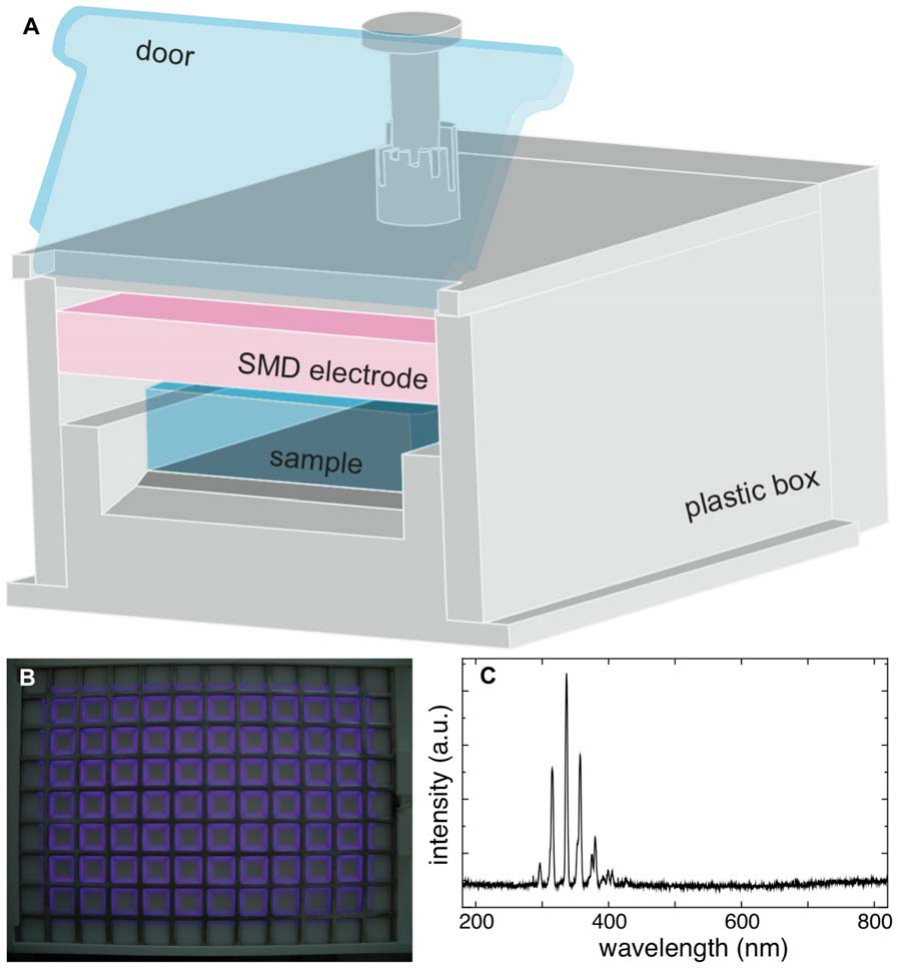
Schematic image of the FlatPlaSter plasma device. a) The FlatPlaSter contains one SMD electrode and the samples (to treat) are placed inside the device below the electrode. In this study the distance between the electrode and the sample surface was fixed at 6 mm. b) left: The plasma discharge is shown. On the mesh-side of the electrode, the SMD plasma was produced. c) right: The spectrum of the SMD plasma observed in front of the electrode with a distance of 6 mm is shown. The UV components in the range between 280–400 nm are mainly produced from nitrogen molecules excited by electrons.

The FlatPlaSter plasma device is a box made out of plastic (Teflon and polyoxymethylene) incorporating an electrode for the plasma production inside as shown in figure 1A. On one side of the box, a door is installed. By closing the door the produced plasma gas cannot escape and therefore is confined inside. This plasma device is designed for the treatment of a 96-well plate (sample), so that the maximum treated area equals 9×13 cm^2^. The plasma electrode is placed above the sample and the distance between the electrode and the sample is adjustable. In our experiments, the distance to the pig skin surfaces was set to 6 mm. By using the ambient air a plasma discharge is ignited with the SMD (Surface Micro-Discharge) plasma technique [19]. The plasma electrode consists of a 0.5 mm thick Teflon plate sandwiched by a brass planar plate and a stainless steel mesh grid (line width 2 mm, opening 10 mm, height 1.5 mm). High sinusoidal voltage of 9 kV_pp_ with 1 kHz in frequency was applied between the brass electrode and the mesh electrode thereby producing the plasma discharges on the mesh side of the SMD electrode as shown in figure 1B. The power consumption for the plasma discharge was approximately 0.02 W/cm^2^ measured with the Lissajous method using a 0.1 µF capacitance.

#### miniFlatPlaSter

**Figure 2:**
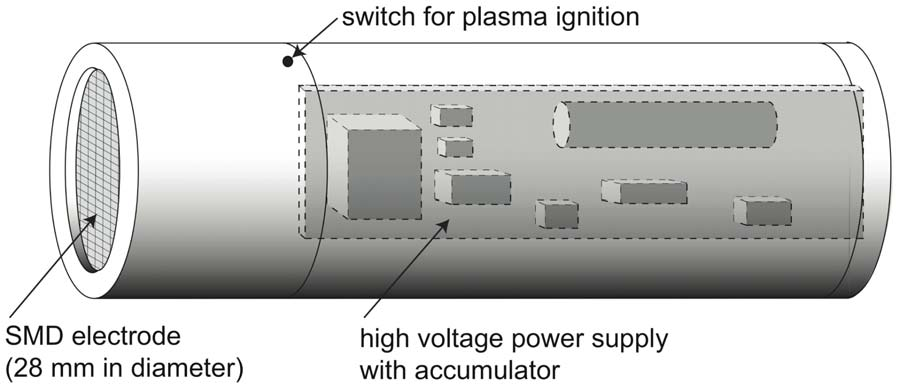
Schematic image of the miniFlatPlaSter plasma device. The portable plasma device is equipped with a high voltage power supply, accumulators, and a surface micro-discharge (SMD) electrode. The SMD electrode consists of a copper foil layer (around 0.2 mm thick), an Epoxy board (1 mm thick), and a stainless steel mesh of 28 mm in diameter. The plasma device was fixed above the *ex vivo* skin embedded in Hepes-Agar.

The plasma device (miniFlatPlaSter), the sketch is shown in Fig. 2, is equipped with a surface micro-discharge (SMD) electrode. This device was designed for portable usage and the high voltage power supply and the accumulators are incorporated. The SMD electrode consists of a copper foil layer (around 0.2 mm thick), a glass Epoxy board (1 mm thick), and a stainless steel mesh. The plasma is ignited on the mesh side of the electrode by applying a high voltage signal between the copper foil and the mesh. The voltage signal is pulse-like with a peak-to-peak voltage of approximately 7 kV and a repetition frequency of approximately 6.75 kHz. The size of the electrode is 28 mm in diameter and the power consumption is approximately 0.5 W/cm^2^.

### Plasma treatment

100 µl of bacteria (∼10^6–7^/ml) was applied to the epidermis of each skin sample. Then the bacteria suspension was spread over the complete skin area of interest using a sterile inoculation loop. The samples were placed under a laminar flow cabinet for 60 min until they were visibly dry. Different plasma treatment times were investigated to determine the antimicrobial decolonization effect.

### Quantification of plasma inactivation efficacy

Post plasma treatment a colony forming unit assay (CFU) was performed to determine the surviving bacteria. A sterile cotton-tipped rod was used to remove bacteria from the epidermis. For each skin sample a cotton-tipped rod was moistened in sampling buffer (0.1% Tween80, 0.0075 M phosphate puffer, pH 7.9) before swabbing the entire surface three times. First the recovery efficacy was tested to establish the range of viable numbers of CFU per ml of untreated skin samples, which was in accordance with already published data [16]. A reproducible correlation between three swabbing steps and the recovery efficiency of bacteria applied to the skin could be evaluated (data not shown). Survived bacteria were detected with the Miles and Misra technique for viable counts [20]. Serially diluted aliquots (20 µl) of treated and untreated samples were plated on Mueller-Hinton agar, and the number of CFU per millilitre was counted after 24 h of incubation at 37°C.

### Histology and histochemistry

Viability of each porcine skin sample was tested by detection of dead cells using the in situ Cell Death Detection Kit according to the manufacturer's protocol (Roche, Penzberg, Germany). A detailed description of the methodology is published elsewhere [16]. Briefly, biopsies of porcine skin samples were embedded in paraffin and sections of 3 µm thickness were cut and mounted on microscope slides (SuperFrost Plus, Menzel Glasbearbeitungswerk GmbH & Co. KG, Braunschweig, Germany). Sections were permeabilised and treated with 3% of hydrogen peroxide to block endogenous peroxidase. Thereafter a TUNEL-reaction was done according to the manufacturer's protocol. Labelling of DNA strand breaks was done by addition of terminal deoxynucleotidyl transferase, which catalyzes polymerization of fluorescein-labelled nucleotides to free 3′OH DNA ends in a template-independent manner. Incorporated fluorescein was detected using a fluorescence microscope (Zeiss Axiotech, Goettingen, Germnay) with an appropriate dual-band filter set (Omega opticals, Brattleboro, VT, USA) for excitation (λ_ex_ = 475±40 nm) and emission (λ_em_ = 535±35 nm). After substrate reaction, the slides were co-stained with DAPI (λ_ex_ = 365±50 nm; λ_em_ = 450±58 nm) to visualize all nuclei. Fluorescence images were superimposed with an image processing program (Image-Pro Plus 5.0; Media Cybernetics, Silver Spring, MD, USA). Negative (no addition of terminal deoxynucleotidyl transferase or the labelled nucleotide mixture of the TUNEL-reaction) and positive controls (addition of DNAse I to induce DNA strand breaks) were included in each experimental set up. The presence of dead cells within the epidermis was defined as green stained nuclei with granular or sometimes fragmented cell nuclei, whereas blue stained cells are negative (DAPI). The number of stained (dead) cells per 100 cells was scored on three individual slides. The number of apoptotic cells was calculated as a percentage of the total number of counted cells as described previously by Schacht *et al.* [21].

### Statistical methods

**Figure 3:**
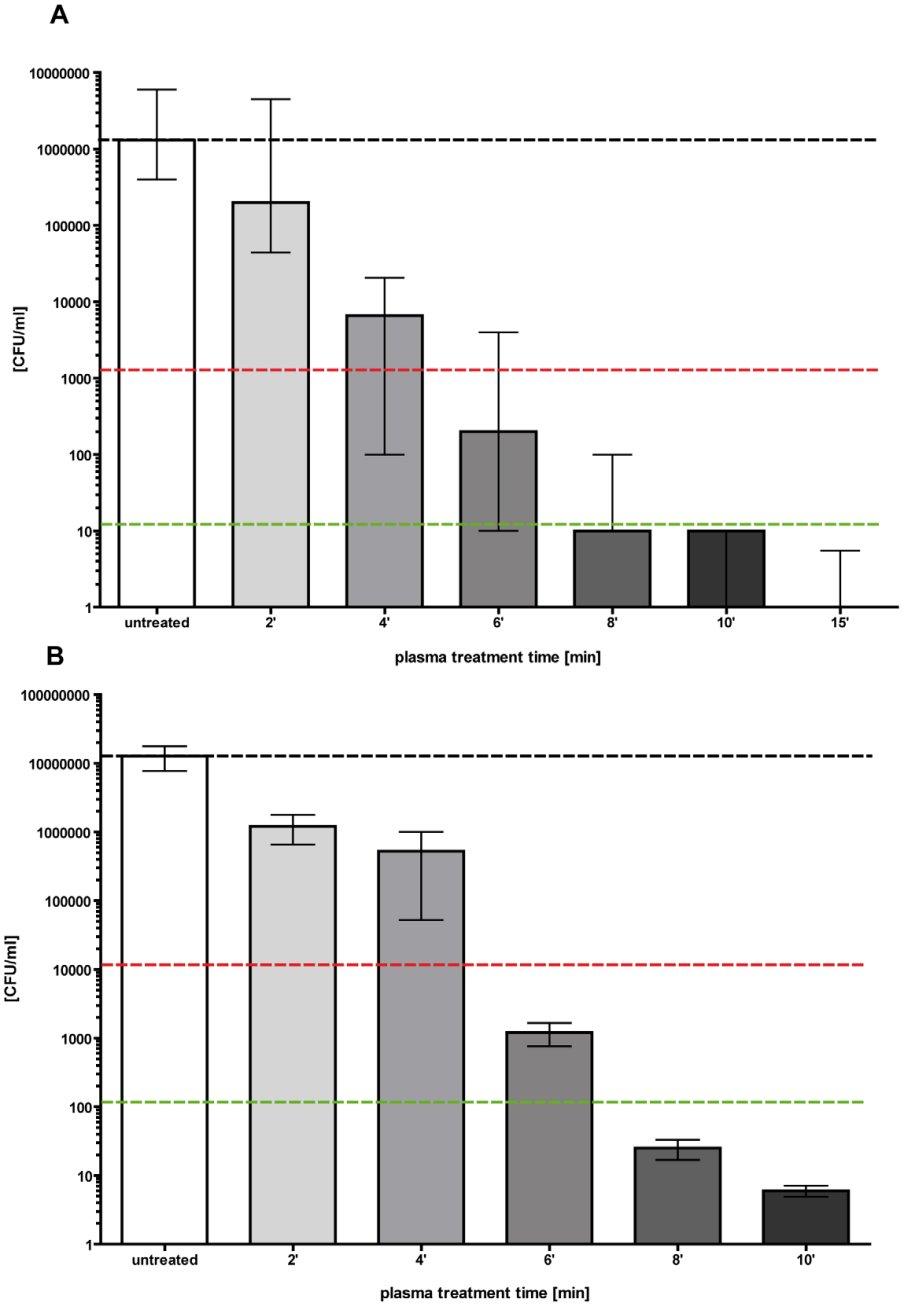
Decolonisation of *S. aureus* and *E. coli* using the FlatPlaSter plasma device. Cold atmospheric plasma treatment of (**A**) *S. aureus* (10^6–7^/ml) (D-value = 3.7 min) and (**B**) *E. coli* (10^6–7^/ml) (D-value = 3.71 min) applied to *ex vivo* skin using the FlatPlaSter device. Different plasma treatment times were tested [sec]. A CFU assay was performed immediately after the plasma treatment. Black dotted line: baseline of viable bacteria per ml; red dotted line: reduction of three log_10_ steps of viable bacteria (99.9%); green dotted line: reduction of five log_10_ steps of viable bacteria (99.999%). (*n* = 6, median ± interquartile range).

**Figure 4:**
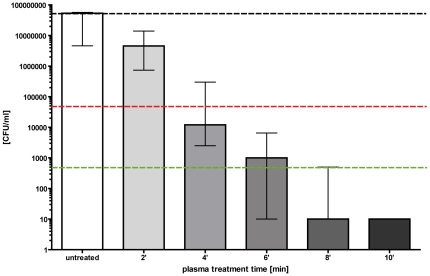
Decolonisation of MRSA using the FlatPlaSter plasma device. Cold atmospheric plasma treatment of MRSA (BAA-44) (10^6–7^/ml) applied to *ex vivo* skin using the FlatPlaSter device. Different plasma treatment times were tested [min]. A CFU assay was performed immediately after the plasma treatment. Black dotted line: baseline of viable bacteria per ml; red dotted line: reduction of three log_10_ steps of viable bacteria (99.9%); green dotted line: reduction of five log_10_ steps of viable bacteria (99.999%). (*n* = 6, median ± interquartile range).

All results are shown as medians, including the 25% and 75% quartiles, which were calculated from the values of at least 3 independent experiments, each experiment was conducted in triplicates, with Prism 4 for Windows (GraphPad Software Inc., San Diego, CA, U.S.A). The calculation (reduction of CFU/ml) was referred to the untreated controls (non plasma treated). In Figs. 3 and 4, medians on or below the dotted horizontal line in red or green represent ≥99.9% efficacy or ≥99.999% of bacteria cell killing corresponding to at least more than three magnitudes or five magnitudes of log_10_ reduction compared to matching untreated controls (non plasma treated). A reduction of at least three magnitudes of log_10_ of viable median numbers of bacteria cells was stated as biologically relevant with regard to the guidelines of hand hygiene [22].

### Calculation of D-value

The death-value (D-value) for each microorganism was defined as the time which is required for one log_10_ or 90% reduction of the initial bacteria number for the specific plasma treatment condition [23].

## Results

### Plasma characteristics

The cold atmospheric plasmas produced in this study deliver various agents to the samples: reactive oxygen and nitrogen species (NO, NO_2_, O_3_, OH, O*, etc.), charged particles (electrons, ions), photons (UV, visible), and heat.

In our experiments, the thermal effect can be ruled out because the increase in the gas temperature was at maximum 4 degrees for 10 minutes of plasma operation.

Due to the distance between the electrode and the sample in our experimental setup (approximately 6 mm), the impact of charged particles (low density around the samples) is very small.

The emitted UV light can be mainly observed from the N_2_ positive system between 280 and 420 nm in wavelength (see spectrum in fig. 1C). In addition peaks in the UVC region resulting from the NO γ system can be detected. The UV power density equals 25 nW/cm^2^. Furthermore it was confirmed that the UV photons (emitted by the plasma) alone did not have any bactericidal effect for a treatment time of up to 120 s. These experiments were carried out by placing a quartz glass plate between the electrode and the respective bacteria samples, so that only the UV light produced by the plasma could pass (data not shown).

Taking all this into account, the main agents which contribute to the inactivation of bacteria in this study are reactive species. As already mentioned, many reactive species are produced by the plasma and all of them can react with the samples. For example, the ozone concentration in the FlatPlaSter (see figure 1A) was at maximum 500 ppm and the concentration of NO_2_ was around a few ppm.

Regarding the two used plasma devices, FlatPlaSter and miniFlatPlaSter, there are no differences in property - both are based on the same SMD technology. Of course, the densities of reactive species, etc. were different because the discharge area, discharge volume, and power input were different.

### Time-dependent cold-atmospheric plasma toxicity against *S. aureus*, MRSA and *E. coli*

First the antimicrobial effect of cold atmospheric plasma was tested against different antibiotic sensitive and resistant bacteria strains using the FlaPlaSter device (fig. 3 & 4). The data indicate that *S. aureus*, MRSA or *E. coli* were killed depending on the plasma treatment time. There was a considerable reduction of CFU ml^−1^ of 3 log_10_ steps for *S. aureus* (D-value = 3.7 min) and *E. coli* (D-value = 3.71 min) after only a plasma treatment time of 6 min using the FlatPlaSter device (fig. 3A/B). By increasing the plasma treatment time up to 15 min the killing efficacy was enhanced. Already a treatment time of 8 min achieved a killing efficacy of ≥5 log_10_ (99.999% of the initial applied bacteria were killed) for both bacteria strains. In order to investigate whether the observed reduction of methicillin-sensitive *S. aureus* was independent of the antibiotic resistance pattern, two different MRSA strains were treated with the FlatPlaSter under conditions identical to those for *S. aureus* and *E. coli*. Similar results were recorded when the MRSA strain BAA-44 was treated with different plasma treatment times (fig. 4). Already a plasma treatment time of 4 min resulted in a killing efficacy of MRSA (BAA-44) of 3 log_10_ steps (99.9%). Increasing the plasma treatment time to 8 min a killing efficacy of 5 log_10_ steps (99.999%) was achieved (D-value = 3.9 min). As expected, the plasma treatment of the second MRSA strain (atcc 43300) resulted in a substantial decrease of CFU ml^−1^ as well (D-value = 3.6 min). The mean reductions in CFU ml^−1^ were 3 log_10_ or 5 log_10_ upon plasma treatment of 4 or 8 min respectively (data not shown).

**Figure 5:**
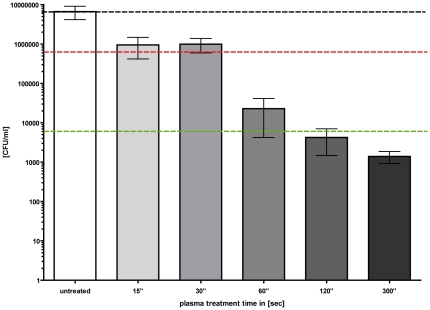
Decolonisation of *S. aureus* using the miniFlatPlaSter plasma device. Cold atmospheric plasma treatment of *S. aureus* (10^7^/ml) applied to *ex vivo* skin using the miniFlatPlaSter device. Different plasma treatment times were tested [sec]. A CFU assay was performed immediately after the plasma treatment. Black dotted line: baseline of viable bacteria per ml; red dotted line: reduction of one log_10_ step of viable bacteria (90%); green dotted line: reduction of three log_10_ steps of viable bacteria (99.9%). (*n* = 3, mean value ± standard deviation).

The second set of experiments was devoted to studying the efficiency of decolonisation of bacteria applied to *ex vivo* skin using the miniFlatPlaSter as a handheld device (fig. 2). Figure 5 shows the decrease of CFU ml^−1^ of *S. aureus* obtained with different plasma treatment times. A decrease of ∼3 log_10_ steps (∼99.9%) of viable *S. aureus* was achieved within 60 sec. Prolonging the plasma treatment to 5 min resulted in a killing efficacy of ≥3 log_10_ steps (≥99.9%) (fig. 5).

### Viability of porcine skin after plasma treatment

**Figure 6:**
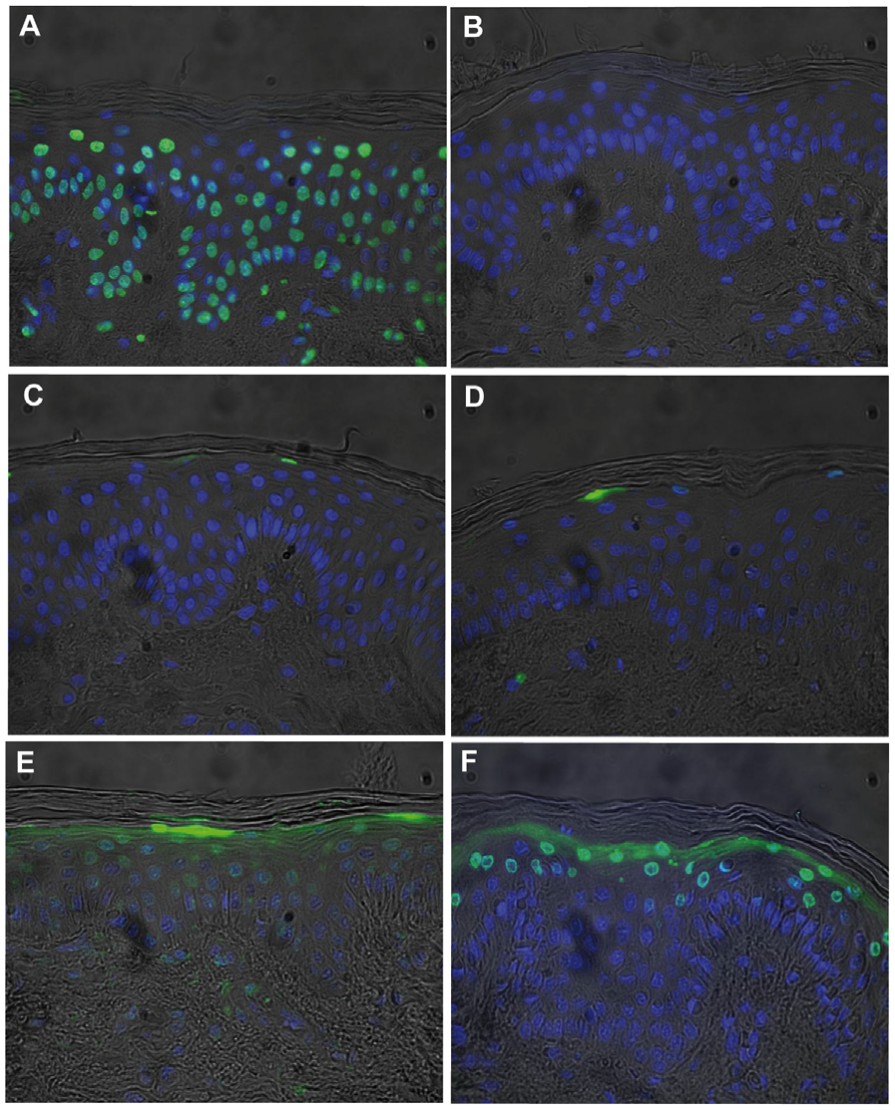
Histological evaluation of *ex vivo* porcine skin after plasma treatment. TUNEL staining of skin sections after the plasma treatment of skin samples inoculated with bacteria. (A) Positive control: incubation with 300 U DNase; (B) negative control; (C) immediately after the 15 min plasma treatment; (D) 24 h after the 15 min plasma treatment; (E) 48 h after the 15 min plasma treatment; (F) 48 h of an untreated skin sample (40×magnifications).

In order to assess the influence of the plasma treatment time on the viability of the ex vivo porcine skin under experimental conditions a TUNEL assay was performed to analyse the number of DNA damaged cells within the epidermis within 48 h after treatment. Normal untreated skin samples embedded in Hepes Agar did not show any morphological changes up to 48 h after the treatment (data not shown). During the entire observation period a total of 12±6% of TUNEL-positive cells was counted within the epidermis of untreated samples. Plasma treatment regardless of the used plasma device and treatment time also showed no increase of TUNEL-positive cells within 24 h (fig. 6). Only a small fraction of positive stained, DNA damaged cells were detected within the upper layers of the epidermis after 48 h, but this was independently when the samples were treated or untreated (fig. 6E and 6F). No TUNEL-positve cells were detected within the dermis.

## Discussion

In this study an *ex vivo* porcine skin model was used to evaluate the antimicrobial decolonisation efficacy of cold-atmospheric plasma against antibiotic-sensitive and resistant bacteria. The results demonstrate that killing of MRSA, *S. aureus* and *E. coli* by cold atmospheric plasma was time-dependent without harming the underlying tissue. Freshly excised porcine skin was used, because it is proposed as a good test model for human skin based on many physiological and anatomical similarities [18]. Histological evaluation of plasma-treated skin samples showed only a slight increase of TUNEL-positive cells within the epidermis, indicating that the porcine skin might be still vital. However 48 hours after the plasma treatment we could detect TUNEL-positive cells in both plasma-treated samples and the untreated controls. This means that the observed damage to the eukaryotic cells is not directly related to the plasma treatment rather than to the total cultivation time of the ex vivo porcine skin. Furthermore cold atmospheric plasma seems not to influence the underlying tissue, because no TUNEL-positive cells were detected at or below the basal membrane or the dermis. Bush and colleagues demonstrated in their studies the feasibility of pig skin as a model substrate for evaluating skin disinfectants [24]. However, in their study skin samples were frozen prior to evaluation of topically applied new antimicrobial agents. We, in this study, used freshly excised skin samples. In spite of this, the results obtained by Bush et al. are more related to the hand-washing technique than to test the efficacy of new antimicrobial techniques using vital skin samples [24]. Recently Messager et al. could demonstrate, when testing several liquid disinfectants that antiseptics were more active in combination with mechanical effects *ex vivo* [25]. The addition of a mechanical effect by drill rubbing of two skin samples against each other produced a significantly greater reduction of the bacterial load [25]. In this study we demonstrated for the first time, that cold atmospheric plasma achieved a killing efficacy of up to 99.999% against topically applied bacteria, inclusively MRSA, via a contactless procedure without damaging the surrounding tissue (Figs. 3, 4 and 5). Then non-linear bacterial reduction with regard to the plasma treatment time may have several reasons:

With the used bacterial concentration of 10^6–7^ per ml and 0.1 ml applied to an area of at maximum 4 cm^2^ we get a bacterial load of 2.5×10^4–5^ per cm^2^ which is in the range of what is found on human skin. For a typical bacterial cross sectional area of 2×10^−8^ cm^2^ this gives a probability of overlap of 5×10^−4^ to 5×10^−3^. This theoretical probability of overlap – assuming a homogeneous distribution - is negligible and so is biofilm formation. However, there could still be concentrations of bacteria in skin folds or follicles. This is something difficult to rule out. Our primary aim in this study was to investigate whether the plasma technique could be useful for the inactivation of an antibiotic-resistant planktonic bacteria strain on skin but some concentration fluctuations cannot be ruled out on “real” surfaces like skin. As we pointed out this may happen even when the bacteria density is low. This could then be one of the reasons why the bacterial reduction rate is not linear.

Furthermore the non-linear reduction can also be explained by the necessity of a certain treshold value: enough reactive species have to be generated by the respective plasma device -above this value bacteria can then be killed very efficiently.

Therefore we can only define an overall treatment time where we achieve a 3 or 5-log_10_ reduction of CFU/ml as described in the Results section.

Successful decolonisation of patients colonised with multi-resistant bacteria is of interest for controlling and preventing bacterial spread in hospital daily routine. Recently Krishna and colleagues reviewed the available literature on the use of disinfectants for MRSA decolonisation. They concluded that the clinical outcome of an octenidine/dihydrochlorine solution for eradication of MRSA varied strongly between 6% and 75% [26]. In a further study of Johnson et al. which examined the successful decolonisation of MRSA by means of an alcohol/chlorhexidine hygiene solution, the consumption of the disinfection solution increased from 5.7 to 28.6 L/1000 bed-days [27]. In another study successful decolonisation was achieved only in patients who completed the full decolonization treatment course for 5 days [28]. The decolonization treatment consisted of mupirocin nasal ointment, chlorhexidine mouth rinse, and full-body wash with chlorhexidine soap. In all these studies the success depends on how many body regions are colonised by MRSA, as well as on the compliance of the treatment protocol by the patients and the health care workers.

This emphasizes the need for the development of additional strategies for the decolonisation of bacteria. Cold atmospheric plasma typically delivers up to 10^15^ active molecules per cm^2^ and second. Here a plasma treatment time of approximately 6 min delivered up to 3×10^9^ active molecules for 1 µm^2^, which were needed to destroy bacteria without any carrier medium. In contrast active ingredients of anti-disinfectants have to be immersed inside an appropriate carrier. When liquids are used for disinfection, the active molecule delivery rate is roughly 10^24^ cm^−2^ s^−1^.

Therefore it could be shown that the plasma treatment is very efficient and fast in comparison with liquid treatment.

It is still under investigation why plasma treatment is so effective. Recently, Kim et al. demonstrated a significant 3.83 log_10_ decrease of CFU of adherent *S. aureus* by employing a plasma discharge in liquid using a reconstructed human skin model [14]. In this study liquid between the area of interest and the plasma device was needed to generate reactive molecules to destroy the bacteria. This means that the plasma device needs direct contact to the area of interest. The advantage of the cold atmospheric plasma treatment, which was used here, is that no direct contact with the sample is necessary to kill the bacteria applied to the stratum corneum. Our hypothesis is that the plasma produced gas forms a “cocktail of reactive species” and due to synergetic properties, the bactericidal effect is very pronounced. There are three principal effects of plasma action: (I) permeabliisation of the cell wall/membrane, leading to leakage of potassium; (II) access of reactive species intracellular to induce oxidative/nitrosative burst; (III) direct chemical reaction to DNA damage. All the reactions depend on the non-equilibrium plasma chemistry (e.g. hydrogen denaturation for membrane permeabilisation, density of ROS and RNS species produced in air and then the reactions inside the cell – e.g. Fenton's reaction as an example, which involves H_2_O_2_). The chemical composition produced by the plasma varies with power and time and therefore might be designable so that no negative impact on eukaryotic cells occurs [29]. In case of the miniFlatPlaSter a larger density of chemically reactive species is produced with higher power input as compared to the FlatPlaSter and because of that the inactivation speed is faster (fig. 5). As described in the Results section both plasma devices used for this study produce - amongst others species - ozone in the ppm region. For sterilization of medical equipment or the *in vivo* treatment of infected skin areas, it is planned to include a filter system using a pump for toxic gas removal, to exchange the gas (air) between the respective plasma treatments and to produce a “sufficiently closed” volume which covers the area of interest. This means that the transmission of possibly toxic components can be avoided. Therefore the plasma is a good medium for energy transfer for inactivating bacteria applied to vital skin.

Overall, the results demonstrate the considerable potential of cold atmospheric plasma in decolonisation of pathogenic bacteria, which were applied to intact skin surfaces *in vitro*. Using two different plasma devices we were able to show, that a bacterial reduction of up to 3 log_10_ steps was achieved for a 60 sec (miniFlatPlaSter)/4 min (FlatPlaSter) plasma treatment; more than a 5 log_10_ reduction was achieved after 8 min (FlatPlaSter) of plasma treatment - without causing any damage to the porcine skin. Although the applicability of this special approach using these prototype devices is too long for hand disinfection at the moment, cold atmospheric plasma might be a powerful tool for topical contact-free use to prevent nosocomial transmission of multiresistant pathogens, like MRSA, because here the full decolonisation treatment course is much longer.
